# Transcriptome signatures in *Helicobacter pylori*-infected mucosa identifies acidic mammalian chitinase loss as a corpus atrophy marker

**DOI:** 10.1186/1755-8794-6-41

**Published:** 2013-10-11

**Authors:** Intawat Nookaew, Kaisa Thorell, Kuntal Worah, Shugui Wang, Martin Lloyd Hibberd, Henrik Sjövall, Sven Pettersson, Jens Nielsen, Samuel B Lundin

**Affiliations:** 1 Department of Chemical and Biological Engineering, Chalmers University of Technology, Gothenburg, Sweden; 2 Department of Microbiology and Immunology, Institute of Biomedicine, Sahlgrenska Academy, University of Gothenburg, Gothenburg, Sweden; 3 National Cancer Center, Singapore, Singapore; 4 Department of Microbiology and Tumor and Cell Biology, Karolinska Institute, Stockholm, Sweden; 5 Genome Institute of Singapore, Singapore, Singapore; 6 Institute of Medicine, Sahlgrenska Academy, University of Gothenburg, Gothenburg, Sweden

**Keywords:** Corpus gastritis, Gastric cancer, Integrated analysis, Acidic mammalian chitinase

## Abstract

**Background:**

The majority of gastric cancer cases are believed to be caused by chronic infection with the bacterium *Helicobacter pylori,* and atrophic corpus gastritis is a predisposing condition to gastric cancer development. We aimed to increase understanding of the molecular details of atrophy by performing a global transcriptome analysis of stomach tissue.

**Methods:**

Biopsies from patients with different stages of *H. pylori* infection were taken from both the antrum and corpus mucosa and analyzed on microarrays. The stages included patients without current *H. pylori* infection, *H. pylori*-infected without corpus atrophy and patients with current or past *H. pylori*-infection with corpus-predominant atrophic gastritis.

**Results:**

Using clustering and integrated analysis, we found firm evidence for antralization of the corpus mucosa of atrophy patients. This antralization harbored gain of gastrin expression, as well as loss of expression of corpus-related genes, such as genes associated with acid production, energy metabolism and blood clotting. The analyses provided detailed molecular evidence for simultaneous intestinal metaplasia (IM) and spasmolytic polypeptide expressing metaplasia (SPEM) in atrophic corpus tissue. Finally, acidic mammalian chitinase, a chitin-degrading enzyme produced by chief cells, was shown to be strongly down-regulated in corpus atrophy.

**Conclusions:**

Transcriptome analysis revealed several gene groups which are related to development of corpus atrophy, some of which were increased also in *H. pylori*-infected non-atrophic patients. Furthermore, loss of acidic chitinase expression is a promising marker for corpus atrophy.

## Background

Chronic infection with the bacterium *Helicobacter pylori* (Hp) can have dire consequences. The majority of infected individuals remain symptom-free, but 10-15% develop peptic ulcers, and 1-3% develop gastric cancer (GC) [1]. Hp-infected subjects are estimated to have up to 12 times increased risk of developing GC, and eradication of *H. pylori* reduces the risk of developing disease [2]. Gastric cancer is today the second largest cause of cancer mortality worldwide with more than 700 000 deaths annually [3].

A hallmark in the progression towards the intestinal type of GC is the presence of atrophic gastritis. It is well established that atrophy of the corpus mucosa, with accompanying loss of parietal cells and thereby decreased acid secretion, is highly associated to GC development. Alongside with corpus atrophy, there is often antralization of the corpus mucosa. This is also termed pseudopyloric metaplasia [4] meaning that the atrophic corpus mucosa attains the general appearance of antral mucosa. Recently, a new lineage of metaplastic cells has gained increased attention - the spasmolytic polypeptide expressing metaplasia (SPEM) cells. These cells develop in the atrophic pits and appear to arise from either a cryptic progenitor cell population in the base of the fundic glands, or from trans-differentiated chief cells [5]. SPEM, which is characterized by strong expression of trefoil factor 2 (TFF2), has been suggested to be present in all atrophy of the corpus [6]. The molecular details of atrophy development, antralization and SPEM, however, are poorly understood and there is a lack of knowledge of the disease progression. Therefore, understanding of the progression towards GC in better detail constitutes an important research area. In the present study, we set out to analyze the genome-wide gene expression of both corpus and antrum mucosa in patients with current or past *H. pylori*-infection and suffering from atrophic gastritis (Atr group), as well as in *H. pylori*-infected patients with non-atrophic gastritis, and patients without current *H. pylori*-infection. The analysis was done using oligonucleotide microarray, followed by detailed systems analysis. This experimental set up, which was based on the disease progression, allowed for the elucidation of the molecular patterns and processes associated with *H. pylori*-induced atrophy in the corpus mucosa.

## Methods

### Patient recruitment and sample collection

The study was approved by the Human Research Ethics Committee at University of Gothenburg and informed oral and written consent was obtained from each volunteer before participation. Patients were selected from a consecutive cohort of patients undergoing gastroendoscopy at Sahlgrenska University Hospital, Gothenburg, during 2007–2009. These patients had been admitted to endoscopy due to dyspepsia, malabsorption or anemia. Patients not suffering from extragastric malignancy or inflammatory disease were carefully grouped into three groups, based on histopathological scoring according to the Sydney system, in combination with *H. pylori* culture and serology results; 1. no current *H. pylori* infection (Hp-); 2. current *H. pylori* infection without corpus-predominant atrophic gastritis (Hp+); 3. current or past *H. pylori* infection and corpus-predominant atrophic gastritis (Atr) (see details in Table 1). For details of sampling procedures and *H. pylori* serology, see Additional file 1.

**Table 1:** Information about of all patients included in the study

**Age**	**Gender**	**Group**	**Analyses performed**^**1**^			**Neutrophils**^**2**^		**Mononuclear cells**^**2**^		**Atrophy**^**2**^		**Intestinal metaplasia**^**2**^	
			**Array**	**qPCR**	**WB**	**Antrum**	**Corpus**	**Antrum**	**Corpus**	**Antrum**	**Corpus**	**Antrum**	**Corpus**
70	Male	Atr		Yes	Yes	0	1	2	2	1	2	0	0
41	Male	Hp+	Yes			2	1	1	1	1	1	0	1
79	Female	Hp+	Yes			2	1	3	2	2	1	1	0
61	Male	Atr			Yes	0	1	0	2	0	2	0	0
62	Male	Atr	Yes	Yes		0	0	1	1	1	3	2	3
78	Male	Hp-	Yes			1	0	1	1	1	1	2	1
68	Male	Hp+		Yes	Yes	0	1	1	1	0	0	0	0
33	Male	Hp+			Yes	0	0	2	1	0	0	0	0
65	Female	Hp+	Yes	Yes		1	1	2	2	0	0	0	0
60	Female	Hp+		Yes	Yes	0	0	1	1	0	0	0	0
63	Male	Hp-	Yes			0	0	2	0	0	0	0	0
63	Female	Atr		Yes		0	1	1	2	0	2	0	0
81	Female	Atr	Yes			0	0	1	2	0	2	0	1
46	Male	Atr	Yes			1	1	2	2	0	2	1	1
71	Male	Hp-	Yes	Yes		0	0	1	0	0	0	0	0
82	Male	Hp-		Yes	Yes	0	0	0	0	0	0	0	0
55	Male	Hp-		Yes		0	0	0	0	0	0	0	0
42	Female	Atr		Yes	Yes	1	3	2	3	0	2	0	0
67	Female	Hp-		Yes	Yes	0	0	0	0	1	0	0	0
74	Male	Hp+		Yes		2	1	2	2	1	1	1	1
56	Female	Hp+			Yes	0	0	2	0	0	0	0	0

^1^The assays performed, using samples from each individual patient, are shown.

^2^Paraffin-embedded sections were analyzed and scored according to the Updated Sydney system (Am J Surg Path 1996:20:1161), where scores may be judged 0 (absent), 1 (mild), 2 (moderate) and 3 (severe).

### Microarray experiment

For the microarray experiments, three patients were randomly selected for analysis from the Hp- and Hp + groups. From the Atr group, the three patients with most progressed lesions according to the histopathology analysis were selected. These three patients had moderate or marked atrophy (score 2 or 3) and mild, moderate or marked intestinal metaplasia (score 1 to 3) in their corpus tissue. The Illumina’s BeadArray technology was used for transcriptome analysis. cRNA amplification and biotin labeling from 500 ng of total RNA were performed using Illumina® TotalPrep RNA amplification kit (Ambion, Inc., Austin, TX). cRNA yields were quantified with NanoDrop® (Thermo Scientific, Wilmington, DE). Labeled cRNAs (1.5 μg) were hybridized to the Human-8 v2 Expression Beadchips (Illumina, Inc., San Diego, CA) then incubating for 19 h at 58°C before being washed and stained following the manufacturer’s protocol. Beadchips were then scanned using the IlluminaBeadArray-Reader scanner. Signal extraction and primary quality control was carried out with BeadStudio 3.1.7 software (Illumina Inc., San Diego, CA).

### Microarray data acquisition and analysis

**Figure 1:**
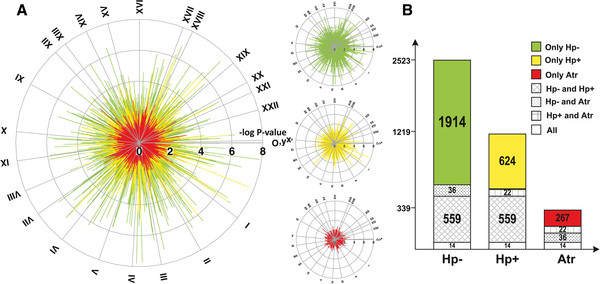
**Differences in global gene expression between antrum and corpus samples in different patient groups. A.** The negative logarithms of p-values for the difference in expression between antrum and corpus tissues are shown as the length of each spike in the circle diagrams (i.e. 8 equals p = 10^-8^), Hp- (green), Hp + (yellow) and Atr (red). All genes in the microarray are shown; the chromosomal location of each gene is indicated by the circle sections in clock-wise direction (chromosome I to XXII). **B.** The number of differentially expressed genes in antrum compared to corpus tissue in Hp-, Hp + and Atr groups, respectively. The sections, and their corresponding numbers, indicate the number of genes significantly different between antrum and corpus in only one patient group (colored sections), or genes for which a difference between antrum and corpus was found in two or three patient groups (patterned sections). The cut-off for inclusion in the figure was a p-value < 0.05 for the comparison between antrum and corpus in each patient group.

**Figure 2:**
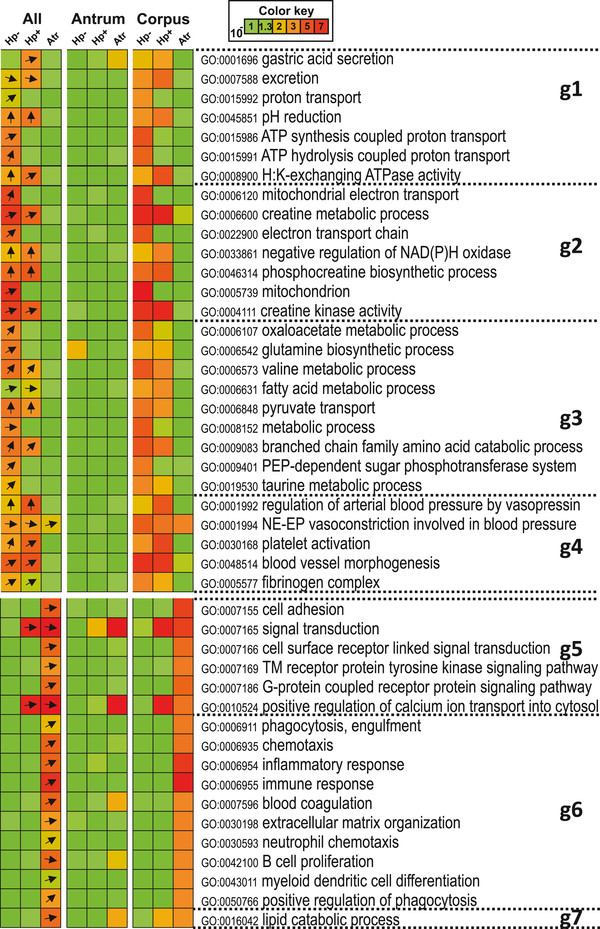
**GO analysis of gene expression differences between antrum and corpus samples in different patient groups.** The ontologies were grouped based on pattern of difference in Hp-, and then further subdivided manually for functional similarity (g1-g7). The “All” column indicates two-tailed analysis, while the results for up-regulation in antrum tissue are shown in the “Antrum” column, and up-regulation in corpus tissue is shown in the “Corpus” column. Arrows indicate the fraction of expression direction of gene member in each ontology (Up arrows = all genes were up-regulated in Corpus, Down arrows = all genes were up-regulated in Antrum). The arrow was drawn when p-value < 0.01. The color-coding indicates the p-values for enrichment of each ontology; i.e. green equals p-values = 10^-1^, and red equals p-values = 10^-8^.

**Figure 3:**
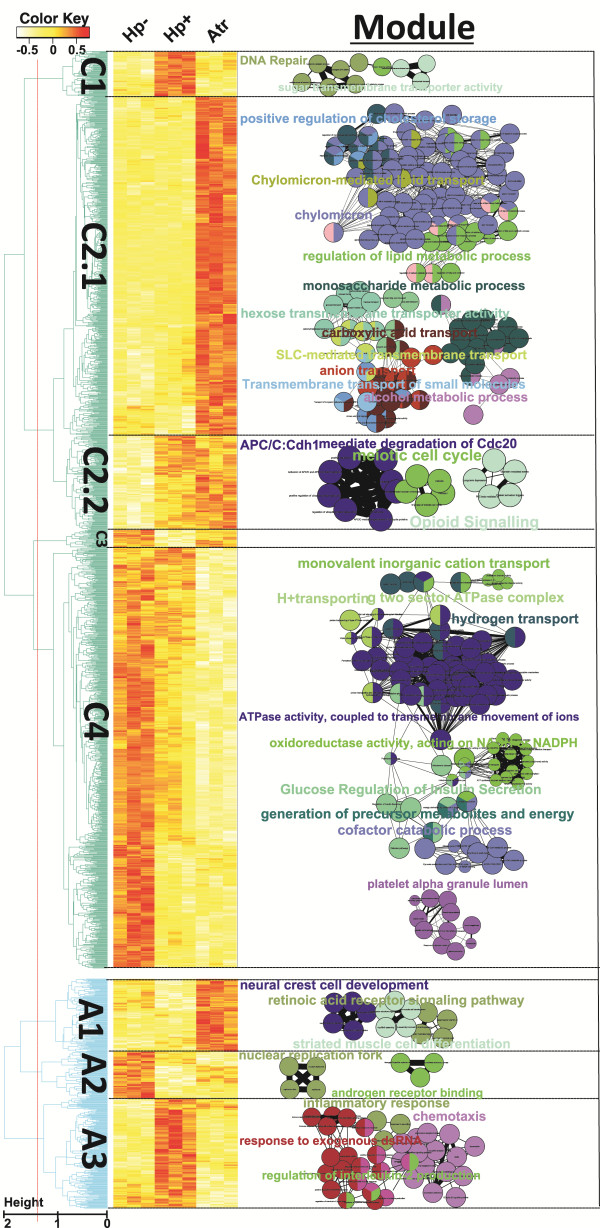
**Hierarchical clustering analysis of corpus and antrum gene expression along the patient groups.** Genes with differential expression across the patient groups (p < 0.001, one-way ANOVA) were clustered. The relative expressions of all clustered genes are shown in heat-map form, where white color indicates down-regulated expression, and red color indicates up-regulated expression. The significantly enriched ontologies are shown in association to each cluster (bubbles). Related ontologies are depicted by the same color, and are connected by lines. The names of the most highly significant ontologies are shown in a large font size. For enlargement of all ontology names, see Additional file 1: Figure S3.

All raw signal intensity files from the BeadStudio were processed together by R software equipped 'lumi’ package [7] using quantile normalization. We used different methods to analysis the microarray data based on different hypotheses. First, the differential gene expressions between the corpus and antrum samples of each group of patients were evaluated by Student’s t test ('limma’ package) [8]. The results are illustrated in global view in Figure 1A and the numbers of differential expressed genes (cutoff p-value < 0.05) of each group are illustrated in Figure 1B. Thereafter, the gene ontology (GO) enrichment of the antrum and corpus samples in each group of patients was revealed by a reporter algorithm [9,10]. The GOs that had enrichment p-value < 0.001 were illustrated in Figure 2 as a heat-map of significant values. Second, the gene expression was analyzed by one-way ANOVA to identify differences among the three patient groups. This was done separately for antrum and corpus samples. The gene expressions that had p-value < 0.001 were further selected for consensus-clustering analysis [11]. Hierarchical clustering of gene expression profiles were performed separately for corpus and antrum samples. The results are presented as a heat-map of gene expression similarity, using a cluster dendrogram height of 1.3. Functional enrichments of each cluster were then evaluated by module enrichment analysis [12] of its gene members, and illustrated using Cytoscape software [13] as illustrated in Figure 3. Third, linear regression analysis was performed over the selected genes based on ANOVA, as described above, to estimate the level of gene expression responses along the three groups of patients. An overview of the analysis layout, and detailed results are provided in Supplementary information (Additional file 1: Figure S1 and Additional file 2).

The microarray results were not subjected to FDR-analysis or multiple-test correction. This is due to the fact that we were limited in the number of well-characterized tissue samples we could obtain from each patient group. However, the key findings in this study were validated with real-time PCR as well as the protein abundance level. The microarray data are deposited and publicly available in Gene Expression Omnibus (GEO; Accession number GSE27411).

### Tissue and cell type specific evaluation of gene clusters

To investigate whether the list of genes up-regulated in atrophic gastritis were enriched in genes with tissue-specific expression patterns, we used a publicly available reference dataset “Human Body Index – Transcriptional Profiling” (GEO-accession number GSE7307). All data were normalized together with the qspline algorithm [14] with signal processing method by Probe Logarithmic Intensity Error [15]. Genes were assayed for tissue specificity compared to small intestine, colon, antrum (“pyloric”), and cardiac/fundus tissue after verifying the validity of the data through hierarchical clustering of the tissues. Genes were analyzed using Wilcoxon rank-sum test and considered tissue specific if they had a p-value < 10^-3^ and a fold change of > 2. The tissue specific genes were then tested for enrichment in the different clusters of Figure 3 using Fisher exact test. The genes in different clusters also tested against the list of genes differentially expressed in IM, SPEM and both IM and SPEM from the recent publication by Lee et al. 2010 [16].

### RT-qPCR assay of selected genes

Selected genes from the microarray analysis were independently validated by RT-qPCR analysis. cDNA synthesis from 600 ng of RNA were performed by Omniscript RT kit (Qiagen, Germany). Each 20 μl RT-qPCR reaction mixture included 2 μl of the cDNA product, 17 μl SYBR Green master mixture (Applied Biosystems) and primers (Additional file 1: Table S1). All assays were performed in technical duplicates using a 7500 Real Time PCR System (Applied Biosystems). Hypoxanthine-phophoribosyl-transferase *(HPRT1),* which has previously been observed to be a good reference gene for normal stomach tissue [17], was used as a reference gene for all assays to normalize expression by the ΔCt method [18].

### Western blot assay of acidic mammalian chitinase protein in stomach

One or two biopsies from antrum and corpus mucosa, respectively, were collected and stored for protein extraction as described above.

At the time of protein extraction, biopsy specimens were incubated in 400 μl PBS, containing 2% saponin, 100 mg/ml soybean trypsin inhibitor, 350 mg/ml phenylmethylsulfonylfluoride and 0.1% bovine serum albumin (all purchased from Sigma-Aldrich, St.Louis, MO, USA), over night at 4°C. The mixtures were then centrifuged at 13000 × g for 10 minutes, and the supernatants were collected and stored frozen at -70°C until used. To ensure equal loading of total protein in the gels, the extracts were run through Zeba Micro Desalt Spin Columns (Nordic Biolab labdesign, Täby, Sweden), whereafter their protein concentrations were determined using BCA Protein Assay reagent (Thermo Fisher Scientific, Rockford, USA). Three μg protein of each lysate was loaded per lane in 4-12% BisTris NuPAGE gels (Invitrogen, Carlsbad, CA) and gels were run in MOPS (3-[N-morpholino]propane sulfonic acid) buffer at 200 V. Proteins were transferred to 0.22 μm nitrocellulose membranes at 30 V using 25 mM Tris–HCl, 192 mM Glycine and 10% methanol for 1 h. Membranes were blocked for 1 h in 2% BSA-PBS/Tween. Rabbit anti-AMCase (ab72309, Abcam, Cambridge, UK) was used as primary antibody, with chrompure Rabbit IgG (Jackson Immuno, Suffolk, UK) as unspecific control. After washes in PBS + 0.1% Tween-20, Goat anti-Rabbit Immunoglobulins-alkaline phosphatase (Southern Biotech, Birmingham, AL) was added and after subsequent washes the blots were developed using 5-bromo-4-chloro-3-indole-phosphate/nitro blue tetrazolium (Southern Biotech, Birmingham, AL). The membranes were scanned with a Geldoc (Bio-Rad Laboratories, Hercules, CA) scanner, and band intensities were analyzed using Quantity One 4.6.1 software (Bio Rad).

## Results

### Confirmation of differences between antrum and corpus samples

We first performed an initial quality check of the microarray data, by investigating the expression of a number or cell-specific genes known to be preferentially expressed either in corpus or antrum tissue. The genes included the corpus specific genes histidine decarboxylase (*HDC*), gastrin receptor (*CCKBR*), H^+^/K^+^ ATPase (*ATP4B*), ghrelin (*GHRL*) and pepsinogen (*PGA5*), and the antrum-specific gene gastrin (*GAST*). The analysis was done using the data from *H. pylori* uninfected (Hp-) individuals. We confirmed a very strong over-expression (10–50 fold) of the corpus-specific marker genes in corpus biopsies using the microarray data, and a more than 800-fold over-expression of the antrum-specific gene gastrin in antrum samples (Table 2).

**Table 2:** Gene expression in antrum and corpus tissue of Hp- individuals, for known antrum- and corpus-specific genes

**Tissue specificity**	**Cell type**	**Description**	**Symbol**	**ID**	**Fold difference**^**1**^	**p-value**^**2**^
**Corpus**	ECL-cell	Histidine decarboxylase	HDC	ILMN_98661	11	1.0 x10^-4^
	ECL/Parietal cell	Gastrin receptor 1	CCKBR	ILMN_9090	15	5.5 x 10^-4^
	Parietal Cell	ATPase H^+^/K^+^ exchanging, beta	ATP4B	ILMN_12353	49	1.6 x 10^-3^
	Parietal Cell	ATPase H^+^/K^+^ exchanging, alpha	ATP4A	ILMN_16471	45	4.5 x 10^-5^
	Parietal cell	Gastric intrinsic factor	GIF	ILMN_18902	17	1.3 x 10^-2^
	A-like/Gr cell	Ghrelin	GHRL	ILMN_19385	10	5.0 x 10^-2^
	Chief cell	Pepsinogen	PGA5	ILMN_23664	48	2.4 x 10^-2^
**Antrum**	G-cell	Gastrin	GAST	ILMN_23553	848	2.8 x 10^-5^

^1^ Fold difference when comparing gene expression levels in corpus vs. antrum for corpus specific genes, and antrum vs. corpus for antrum-specific genes.

^2^ Antrum and corpus expression was compared using Student’s t-test; only values ≤ 0.05 are shown.

### Depletion of differential gene expression between antrum and corpus mucosa in atrophic gastritis

In order to understand the characteristics of the atrophic corpus mucosa, we applied a global analysis of differential gene expression between antrum and corpus samples of the three patient groups. This analysis demonstrated that the most strongly significant differences between antrum and corpus gene expression are found in the Hp- group, followed by the Hp + gastritis and atrophy groups (Figure 1A). There was also a dramatic reduction in the number of differentially expressed genes in antrum vs. corpus samples from corpus atrophy patients: 90% and 75% reduction compared to Hp- and Hp + groups, respectively (Figure 1B). Among these genes, around equal numbers were over-expressed in antrum and in corpus. Over-expressed genes in corpus amounted to 54%, 60% and 41% of the differentially expressed genes in the Hp-, Hp + and Atr groups, respectively. Thus, our data thus demonstrates molecular evidence for a strong similarity of global gene expression between corpus and antrum tissue of patients suffering from corpus atrophy. This is also supported by the result of a transcriptome based hierarchical clustering of samples, as shown in Additional file 1: Figure S2.

### Corpus-specific genes in the un-infected stomach include genes related to acid secretion, energy metabolism, and blood vessel activity

We then performed an integrated analysis to assess over-representation of different biological processes among the genes differentially expressed in antrum compared to corpus mucosa. This analysis demonstrated that the absolute majority of gene ontologies coupled to differential expression between antrum and corpus mucosa in Hp- individuals were associated with genes over-expressed in the corpus tissue (Figure 2). Thus, in all 45 ontologies there was significant enrichment of corpus over-expressed genes, and in 7 ontologies there was also enrichment of over-expression of genes in the antrum tissue.

Groups g1 through g4 (Figure 2) encompass ontologies over-expressed in corpus mucosa in the Hp- group. Therefore, these groups yield important information about the functions of genes specific for the corpus mucosa of Hp- non-atrophic stomach. G1 includes gene-groups related to gastric acid secretion and H^+^/K^+^-ATPase activity. A large part of the corpus-specific gene ontologies concern energy turnover and mitochondrial function (g2) as well as amino acid and lipid metabolism (g3). The g4 group indicates that an important function of the corpus mucosa is related to blood vessels and blood clotting. In this group, genes related to fibrinogen complex, platelet activation and blood vessel growth were found (see Additional file 1: Table S2 for details). This is illustrated most clearly by *FGA* and *FGB,* which encode the alpha and beta chains of fibrinogen, respectively. These genes were both among the 15 most highly over-expressed in the corpus compared to antrum of Hp- patients (20- and 31-fold over-expression, respectively). High *FGA-*expression in the corpus mucosa is confirmed by the Human Protein Atlas [19], where very strong patchy staining of FGA is observed in subepithelial cells of the foveolar region, while FGB is stained strongly by both epithelial and subepithelial cells (Additional file 1: Figure S3). The majority of gene ontologies associated to over-expression in corpus atrophy mucosa are related to immune response and inflammation (Figure 2; g6). There were, however, no differences in either active or chronic inflammation scores between the Hp + and Atr groups (Table 1), which indicate that the inflammatory association detected by the global analysis is related to more subtle inflammatory changes in the corpus atrophy mucosa.

### The similarities of gene expression between antrum and corpus mucosa in atrophic gastritis is caused by transcriptional loss of corpus-specific gene groups

In agreement with data shown in Figure 1, the gene ontology (GO) analysis revealed a relative down-regulation of a large number of genes in the atrophic corpus mucosa with the loss of nearly all corpus-specific gene ontologies in the corpus atrophy group (Figure 2, g1 through g4). Strikingly, corpus-specific gene groups related to the acid secretion and the presence of parietal cells showed no difference between antrum and corpus samples of atrophy patients.

To enable a more detailed comparison between the different patient groups, an analysis of gene expression was performed using hierarchical clustering. While the previous analysis (Figures 1 and 2) compared differences between antrum and corpus samples within individual patients, the hierarchical clustering approach compared the gene expression of antrum and corpus samples separately, across the different patient groups. While only 356 genes were captured by the ANOVA cut-off (p-values < 0.001) in antrum samples, there were 1395 differentially expressed genes in the corpus samples. A clustering dendrogram clearly further divided the expression pattern into 4 and 3 clusters for corpus and antrum samples, respectively (Figure 3).

One of the major corpus gene groups (C4; containing 640 genes) contained the genes whose expression was characterized by loss of expression in the atrophy patients (Figure 3). Analysis of enriched functions in C4 revealed that in similarity to the g1-g4 groups from the integrated analysis (Figure 2), the C4 cluster contains an over-representation of genes related to parietal cells including acid secretion and energy metabolism, as well as genes related to blood vessels and platelet activation (Figure 3 – “bubbles”; for details see Additional file 1: Figure S3). These results confirm that the similarities between antrum and corpus samples of atrophy patients (Figure 2) is due to diminished expression of corpus-specific genes in the atrophic corpus, and not to up-regulation of corpus-related genes in the antrum samples of atrophy patients. To address this issue further, the gene clusters were analyzed for enrichment compared to publicly available datasets. This showed that C4 genes, which are down-regulated in the corpus mucosa of atrophy subjects, are indeed strongly associated with expression in healthy corpus mucosa (p = 7.4 × 10^-6^; Table 3), while A1 genes, which are up-regulated in the antrum tissue of atrophy subjects, are not significantly associated with expression in normal corpus mucosa (p =1; Table 3). Taken together, this shows that the majority of corpus-specific genes and gene groups are down-regulated in the atrophic corpus tissue, which leads to a molecular antralization of the corpus mucosa in corpus-atrophy subjects.

**Table 3:** Gene cluster enrichment (p-values) of tissue-specific genes, as well as of IM and/or SPEM related genes

**Gene list**	**Gene cluster**							
	**C1**	**C2.1**	**C2.2**	**C3**	**C4**	**A1**	**A2**	**A3**
**Cardiac/Fundic**^**a**^	0.92^c^	0.92	0.92	1	**7.42 x10**^**-06**^	1	1	0.92
**Pyloric**^**a**^	1	0.68	1	1	0.10	1	1	0.68
**Small Intestinal**^**a**^	1	**1.81 x10**^**-26**^	**2.57 x10**^**-02**^	1	0.82	0.82	1	0.23
**Colonic**^**a**^	1	**8.78 x10**^**-12**^	**7.67 x10**^**-03**^	1	1	0.72	1	0.35
**IM up**^**b**^	1	**1.96 x10**^**-111**^	**1.21 x10**^**-07**^	1	1	0.97	1	1
**IM/SPEM up**^**b**^	1	**5.25 x10**^**-04**^	**6.16 x10**^**-07**^	1	1	1	1	1
**IM down**^**b**^	1	1	1	1	**5.86 x10**^**-37**^	1	0.99	1

^a^P-values less than 0.05 are displayed in bold text. The gene lists were obtained from a publicly available reference dataset “Human Body Index – Transcriptional Profiling”.

^b^The gene lists were obtained from [16].

^c^p-value.

### Detailed molecular evidence for simultaneous IM and SPEM in atrophic corpus tissue, as well as for SPEM development in non-atrophic *H. pylori* infected corpus tissue

In addition to a decrease of a substantial number of corpus-specific genes in atrophic mucosa, there was an almost equally large set of genes that were markedly up-regulated in the corpus tissue of atrophy patients. These genes grouped into the C2.1 cluster, which contains 442 genes (Figure 3). In addition, there were 217 genes that were up-regulated in both atrophic corpus mucosa and Hp + non-atrophic corpus mucosa (C2.2 cluster). A further analysis of C2.1 revealed that a proportion of C2.1 genes were antrum-specific. Using the non-conservative cut-off of p < 0.05 used in the initial differential analysis (Figure 1), 18% of the C2.1 genes were significantly over-expressed in antrum compared to corpus samples of Hp- subjects. Thus, 80 of the genes up-regulated in corpus atrophy mucosa were antrum-specific. An example of such a gene was gastrin (GAST), which showed a strongly increased expression in corpus mucosa of the Atr group compared to the Hp- group and Hp + group (253 fold difference; p = 2.68 × 10^-4^ and 221 fold-difference; p = 2.68 × 10^-4^ respectively).

The functional analysis of C2.1 genes revealed that the majority of ontologies in this cluster were related to functions of the intestinal epithelium, including lipid, cholesterol and sugar transport and metabolism (Figure 3). Indeed, enrichment analysis of a publicly available dataset, GSE7307, demonstrated that there was a robust enrichment of small intestinal-specific genes in the C2.1 cluster (p = 2 × 10^-26^; Table 3). This strongly indicated that the C2.1 cluster was related to IM development in the atrophic mucosa, which agreed with the fact that the three atrophy patients analyzed by the microarray exhibited at least mild IM of the corpus according to histopathology assessment (Table 1). To further investigate the relation to IM, we performed an additional enrichment analysis compared to a recently published dataset of IM as well as SPEM marker genes, obtained by microarray analysis of microdissected human SPEM and IM tissue [16]. Using this data, we observed that the C2.1 cluster exhibited an extraordinary enrichment of IM up-regulated genes (p = 2 × 10^-111^, Table 3). Importantly, there was also a significant enrichment of genes observed to increase in both IM and SPEM tissue (p = 5 × 10^-4^), indicating that the antralization of the corpus was associated with SPEM. Enrichment analysis of the C2.2 cluster, which contained genes increased in the corpus mucosa of both Hp + and Atr groups, revealed an equally high association to genes up-regulated in both SPEM and IM (p = 6 × 10^-7^), as with genes increased in IM alone (1 × 10^-7^, Table 3), demonstrating a stronger association to SPEM genes compared to the C2.1 cluster. Furthermore, 43% of the genes in C2.2 were antrum-specific, as determined by a p-value cut-off of 0.05 for increased expression in antrum tissue of Hp*-* patients. The 5 genes of the C2.2 cluster with the highest antrum-specificity in uninfected mucosa were *MUC17*, *CEACAM6*, *CLDN7*, *SLC6A14* and *XDH* (55-fold, 27-fold, 24-fold, 15-fold and 11-fold over-expression in normal antrum vs corpus, respectively). Some of the gene groups most highly associated with the C2.2 cluster are related to cell cycle regulation (Figure 3 “bubbles”), which shows that even in the pre-atrophic Hp + corpus, there are alterations in proliferative regulation, which may be involved in SPEM development. Strikingly, there was a strong association with DNA repair genes within the C1 cluster, i.e. genes up-regulated in Hp + corpus and then again decreased in the atrophic corpus mucosa. Thus, while proliferative regulation was altered in both non-atrophic and atrophic corpus mucosa, DNA repair genes were increased only in the non-atrophic tissue.

### Analysis of corpus-specific genes lost in the atrophic mucosa reveals acidic mammalian chitinase as a signature for atrophy

In order to search for molecular markers for corpus atrophy, we further investigated which genes were most strongly down-regulated in the corpus mucosa of atrophy patients, by performing a regression analysis of corpus gene expression in the three patient groups (Table 4). This analysis identified the genes which exhibited progressive down-regulation from Hp- individuals to the Hp + and then Atr groups. The gene showing the strongest down-regulation of corpus gene expression in Atr patients was **acidic mammalian** chitinase (*AMCase*), which is an enzyme that degrades chitin under acidic conditions. This gene was also the second most strongly over-expressed gene in the normal corpus mucosa compared to in normal antrum (90-fold difference in the microarray, Table 4). The validity of the regression is shown by the fact that *ATP4B* also was among the top genes (Table 4). Other genes of high interest were chordin (*CHRD*) which is known to be involved in cellular differentiation and development, as well as fibrinogen as mentioned above.

**Table 4:** The genes showing the highest down-regulation of corpus expression in patients with corpus atrophy (ranked by regression coefficients)

**ID**	**Symbol**	**Description**	**Corpus**		**Antrum**		**Fold change**^**b**^			**Cluster**^**c**^
			**B**^**a**^	***R***^***2***^	**B**^**a**^	***R***^***2***^	**Hp-**	**Hp+**	**Atr**	
ILMN_25453	AMCase	chitinase, acidic, transcript variant 1	-3.66	0.69	-0.74	0.04	93	97	2	C4
ILMN_12978	MRGPRD	MAS-related GPR, member D	-3.32	0.71	-0.59	-0.04	103	100	2	C4
ILMN_27134	CHRD	chordin, transcript variant 2	-3.25	0.69	-0.87	0.13	69	65	3	C4
ILMN_24872	AMCase	chitinase, acidic, transcript variant 2	-3.24	0.69	-0.65	0.11	37	31	1	C4
ILMN_16471	ATP4A	ATPase, H+/K + exchanging, alpha polypeptide	-3.03	0.87	-0.78	0.10	46	37	2	C4
ILMN_21851	AQP4	aquaporin 4, transcript variant b	-2.80	0.85	-0.74	0.14	27	25	2	C4
ILMN_23805	AGXT2L1	alanine-glyoxylate aminotransferase 2-like 1	-2.77	0.93	-0.48	0.10	27	9	1	C4
ILMN_11251	PTGER3	prostaglandin E receptor 3, transcript variant 9	-2.67	0.85	-0.58	0.07	27	15	2	C4
ILMN_12353	ATP4B	ATPase, H+/K + exchanging, beta polypeptide	-2.60	0.70	-1.28	0.13	49	46	8	C4
ILMN_11182	FGA	fibrinogen alpha chain , transcript variant alpha	-2.40	0.90	-0.54	0.10	20	12	2	C4
ILMN_13882	FGB	fibrinogen beta chain	-2.39	0.89	-0.26	-0.03	31	3	2	C4
ILMN_17569	OGG1	8-oxoguanine DNA glycosylase, transcript variant 1b	-2.34	0.66	-0.30	-0.02	17	17	1	C4
ILMN_10755	CKB	creatine kinase, brain	-2.29	0.88	-0.76	0.39	14	9	2	C4
ILMN_13759	GPR30	G protein-coupled receptor 30, transcript variant 1	-2.28	0.73	-0.51	0.08	19	12	2	C4
ILMN_20448	CPA2	carboxypeptidase A2 (pancreatic)	-2.21	0.65	-0.38	-0.09	47	26	4	nc
ILMN_20479	SIGLEC11	sialic acid binding Ig-like lectin 11	-2.20	0.91	-0.38	0.04	13	8	1	C4
ILMN_23656	SH3GL2	SH3-domain GRB2-like 2	-2.18	0.78	-0.26	-0.07	17	12	1	C4
ILMN_28182	FLJ35258	hypothetical protein 284297	-2.18	0.76	-0.38	0.47	16	7	1	C4
ILMN_28694	CDH2	cadherin 2, type 1, N-cadherin (neuronal)	-2.17	0.86	-0.20	-0.06	16	7	1	C4
ILMN_5239	FNDC5	fibronectin type III domain containing 5	-2.13	0.88	-0.37	0.29	12	4	1	C4
ILMN_19137	MFSD4	major facilitator superfamily domain containing 4	-2.09	0.86	-0.49	0.16	13	9	1	C4
ILMN_6662	TRIM50	tripartite motif-containing 50	-2.05	0.84	-0.24	-0.07	16	13	1	C4
ILMN_16766	ZNF533	zinc finger protein 533	-2.04	0.85	-0.37	0.07	12	7	1	C4
ILMN_27352	CLIC6	chloride intracellular channel 6	-2.02	0.64	-0.40	-0.05	20	11	2	nc
ILMN_4712	SERPINA5	serpin peptidase inhibitor, clade A, member 5	-2.02	0.88	-0.40	-0.09	14	1	2	C4
ILMN_15602	CLCNKA	chloride channel Ka	-2.01	0.88	-0.28	-0.06	14	8	1	C4
ILMN_19114	DNER	delta-notch-like EGF repeat-containing transmembrane	-2.01	0.86	-0.54	0.22	12	5	2	C4

The goodness of fits were also reported by ***R***^***2***^;

^a^Regression coefficient. Linear regression was performed along the three patient groups (Hp-, Hp + and Atr).

^b^Corpus vs. antrum gene expression fold changes of the individual patient groups.

^c^The result from clustering analysis; nc = not included in clustering analysis.

**Figure 4:**
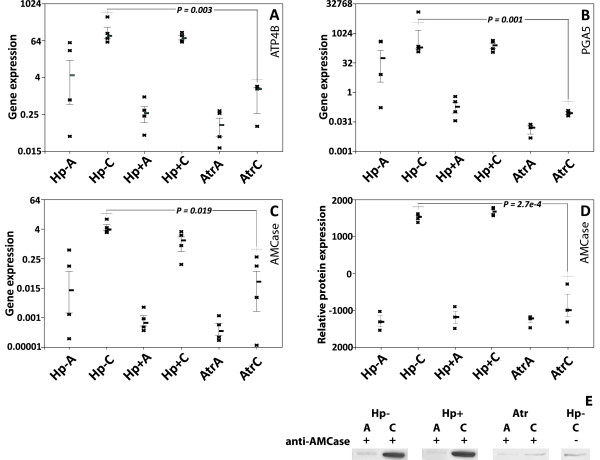
**Gene and protein expression in antrum and corpus mucosa. A, B** and **C**: RT-qPCR verification of H^+^/K^+^ ATPase, *ATP4B***(A)**, pepsinogen, *PGA5***(B)** and acidic chitinase, *AMCase***(C)** versus the reference gene *HPRT* is shown. **D** and **E**: AMCase protein levels analysed with Western Blot. Band intensities were normalized against isotype control antibody **(D)**. A representative blotting image is shown **(E)**; the full image is shown in Additional file 1: Figure S4. The deduced molecular mass of the full length AMCase is 49.9 kDa. Each dot represent one patient sample. The median level is indicated with a horizontal bar, and the interquartile range indicated. Results from both antrum and corpus mucosa from the three patient groups (Hp-, Hp+, Atr) are shown **(A** = antrum; **C** = corpus**)**.

We performed RT-PCR analysis of *AMCase* expression in order to confirm results obtained by the microarray analysis. RT-PCR confirmation was also performed for the *ATP4B* gene, as well as for *PGA5*, which are both known to be down-regulated in atrophic corpus tissue. RT-PCR analysis indeed confirmed that *AMCase* expression was down-regulated, as was expression of *ATP4B* and *PGA5* (Figure 4). To further verify the loss of *AMCase*, we performed Western blot analysis of tissue lysates from antrum and corpus tissue from a number of patients of the three groups. This showed that there was an almost complete loss of *AMCase* in the atrophic corpus mucosa, confirming that loss of *AMCase* expression is a signature for atrophy. On the individual patient level, there was a very strong correlation (r^2^ = 0.92; p < 0.0001) between mRNA and protein levels, supporting the specificity of the western blot assay used (Additional file 1: Figure S5).

## Discussion

Previously, only a limited number of studies have been performed to assess the influence of *H. pylori* infection on the global gene expression pattern in the human stomach [20-22]. However, to the best of our knowledge, this is the first study that uses systems biology tools to analyze the gene expression profile of human atrophic gastritis associated with *H. pylori* infection.

The enrichment analysis of global gene expression differences between antrum and corpus mucosa of Hp- patients confirmed to a large extent previous knowledge on physiological differences. Several of the gene ontologies associated to over-expression in corpus compared to antrum mucosa are directly related to the key function of the corpus, acid secretion (Figure 2, g1). Furthermore, a large number of corpus-associated ontologies are linked to energy turnover and mitochondrial function (Figure 2, g2). This is due to large energy requirements for the fueling of acid secretion, and is consistent with previous results showing a higher respiratory capacity of corpus compared to antrum mucosa [23]. In fact, it has been estimated that as much as 30–40% of the parietal cell cytoplasmic volume is occupied by mitochondria [24,25]. The corpus-associated ontologies related to blood vessel activity is most likely also linked to the metabolic demands [26] and animal studies have shown that ischemia of the corpus tissue leads to rapid necrosis due to the high metabolic rate and relative lack of ability to use glycolysis [27,28].

The down-regulation of *FGA*, *FGB* and other genes involved in blood clotting and platelet activation in the corpus mucosa of the Atr group suggest a dysregulation of fibrinogen expression in these patients, which could impair the protection against influx of harmful substances such as carcinogens into damaged corpus mucosa [29,30]. This may influence the risk of developing severe gastric diseases such as peptic ulcer or GC and future investigations of this are warranted.

The transcriptome analysis revealed a 90% depletion of differences in gene expression between antrum and corpus tissue in patients suffering from atrophic corpus gastritis. This was due to antralization of the corpus mucosa at a global gene expression level – the gene expression pattern in corpus tissue of atrophy patients was largely similar to the pattern of antrum mucosa. Antralization of the corpus is a well-known phenomenon associated to atrophic gastritis [31]. However, this phenomenon has previously mainly been described morphologically and not at the transcriptome level as done in the present study. It is striking to note that antralization of the corpus involves differential expression of more than 2000 genes (Figure 1B).

In addition to the loss of corpus-specific genes leading to antralization of the corpus mucosa, there is also a large set of genes that are up-regulated in corpus biopsies of atrophy patients, which is illustrated by the C2.1 cluster (Figure 3). The majority of the 442 C2.1 genes (> 80%) are genes not normally over-expressed in antrum mucosa but contained genes expressed in the small intestinal tissue. This is shown by gene ontology analysis, which revealed a strong enrichment of intestinal-related gene functions among C2.1 genes (Figure 3). It also agrees with previous observations of similar metabolism in IM as in jejunal mucosa [32]. Furthermore, the intestinal identity of the C2.1 cluster is clearly shown by its very strong association (p = 2 × 10^-26^) to intestinal specific genes extracted from a public dataset (Table 3). In addition, the C2.1 genes were close to identical (p = 2 × 10^-111^) to a list of IM-related genes, obtained using laser-capture microdissection of IM mucosa [16]. The gastrin gene (GAST) was also among the C2.1 cluster genes with the most highly up-regulated expression in atrophic corpus mucosa. This is clearly not a gene related to IM development, but was the one gene with the highest over-expression in healthy antrum vs. corpus tissue on the array (847-fold difference). Interestingly, the expression of GAST increased 240-fold in corpus mucosa of Atr group compared to the rest of the groups. This strongly indicates *de-novo* expression of GAST in corpus tissue and agrees well with a previous report showing that gastrin production is highly associated with antralization of the incisura angularis in Hp + individuals [33].

Several studies have revealed that the process of antralization is characterized by a novel metaplastic transformation of cells in the corpus glands. This distinct novel cell type has been termed SPEM [34]. The presence of SPEM in the corpus atrophy samples is confirmed by strong association of C2.2 to IM and/or SPEM-related genes relative to IM only genes (p = 6 × 10^-7^ and 1 × 10^-4^, respectively; Table 3). Interestingly, the genes of the C2.2 cluster are up-regulated in corpus tissue of both Hp + and Atrophy groups, suggesting that SPEM-associated genes are up-regulated in the corpus mucosa in Hp + patients even in the absence of corpus-predominant atrophy. It could be argued that these findings may be due to misclassification of the patients, due to patchy distribution of atrophy and the fact that histology assessment and microarray analysis were performed on separate biopsies. However, analysis of the data from the microarray concerning individual genes known to be down-regulated in the atrophic corpus tissue clearly demonstrates prominent corpus atrophy in the Atr group but not in the Hp + group. For example, the expression of *ATP4B* was 46-fold and 36-fold higher in corpus than antrum tissue in Hp- and Hp + groups, while only 2-fold higher in the Atr group.

It is striking to note that out of the 217 genes of the C2.2 cluster, 43% were antrum-specific in Hp- patients. Thus, the global transcriptome change reflected in cluster C2.2 is also strongly associated with antralization, and is likely not confined to changes associated only with the SPEM cell lineage.

The clustering analysis revealed the interesting fact that while proliferation and DNA repair genes both are up-regulated in the Hp + corpus mucosa (Figure 3; clusters C1 and C2.2), proliferation genes but not DNA repair genes are up-regulated in atrophic corpus gastritis. We hypothesize that this gene expression pattern leads to an increased occurrence of mutations in the atrophic corpus mucosa and may therefore be an important factor leading to the increased GC risk associated with atrophic gastritis. Interestingly, the novel atrophy model induced by activation of cre recombinase, shows that corpus epithelial cells are especially susceptible to DNA damage [35].

To find genes that may be used as novel molecular markers for corpus atrophy, we analyzed the most strongly down-regulated genes in the corpus mucosa of the Atr group. One striking finding from the analysis of genes down-regulated in corpus atrophy is *CHRD*. This gene exhibited a 30-fold relative reduction in expression in the atrophic corpus mucosa compared to Hp- and Hp + individuals (Table 4). Interestingly, CHRD is a potent negative regulator of bone morphogenic protein (BMP) signaling [36], which is of great importance for the development and differentiation of gastric epithelial cells [37]. Furthermore, mouse studies have shown that loss of BMP signaling can lead to GC development [38]. Taken together, this shows that the role of CHRD in the development of atrophy and GC in humans deserves further study.

The top gene in this list (Table 4) was **acidic mammalian** chitinase, *AMCase*. We confirmed the mRNA and protein expression of AMCase in normal corpus mucosa, and its strong down-regulation in atrophic corpus tissue (Figure 4). AMCase is a chitin-degrading enzyme which is active under acidic conditions, and has been shown to be expressed in chief cells of mammals [39,40]. In contrast to our results, two recent studies described very low or absent levels of AMCase mRNA and/or protein in human stomach [41,42]. However, the mRNA tested in at least one of those studies was from antrum tissue [42], and we indeed show that AMCase is expressed only in the corpus (Figure 4). Furthermore, different antibodies were used for the protein assays in our study and the conflicting study, which may explain the different protein results obtained. The specificity of our western blot assay is supported by a very strong correlation between mRNA and protein levels (r^2^ = 0.92; Additional file 1: Figure S5).

AMCase has been extensively studied in pulmonary tissue where it protects epithelial cells from FasL- and growth factor withdrawal-induced apoptosis. This autocrine or paracrine pro-survival effect is associated with the PI3K/Akt pathway and is independent of its chitinase activity [43]. It has also been shown to play an immune-modulatory role both through its chitinolytic effects but also by stimulating Th2-cells in the pathogenesis of asthma [44]. The loss of chitinase in corpus atrophy might lead to an increased sensitivity to apoptosis and a more Th1-oriented response to *H. pylori* infection, leading to increased tissue damage and severity of disease.

## Conclusions

In conclusion, global transcriptome analysis clearly showed that antralization of the corpus mucosa in atrophic gastritis due to *H. pylori* infection is associated with lost expression of corpus-related gene groups, such as genes related to acid secretion, energy metabolism and blood clotting. In parallel with antralization, corpus atrophy is also associated with increased expression of genes related to inflammation and cell signaling. Furthermore, we propose that loss of expression of **acidic mammalian** chitinase in the corpus tissue may be used as a novel molecular signature for atrophic gastritis.

## Competing interests

The authors declare that they have no competing interests.

## Authors’ contributions

IN performed bioinformatics and statistics analyses and drafted the manuscript. KT performed RT-PCR and WB assays, performed bioinformatics and statistics analyses and drafted the manuscript. SW performed the microarray analysis. KW performed RT-PCR analyses and WB assays. HS, JN and SL were involved in critical revision of the manuscript. All authors contributed to the design of the study, and they all read and approved the manuscript.

## Pre-publication history

The pre-publication history for this paper can be accessed here:

http://www.biomedcentral.com/1755-8794/6/41/prepub

## Supplementary Material

Additional file 1 Contains supplementary materials and methods in addition to supporting data figures. Click here for file

Additional file 2 Contains an excel-formatted table showing the main statistical analyses and the clustering results for each individual gene analyzed. Click here for file
