# Supplementary material for: Transcriptome signatures in Helicobacter pylori-infected mucosa identifies acidic mammalian chitinase loss as a corpus atrophy marker
Source: BMC Med Genomics. 2013 Oct 11;6:41. doi: 10.1186/1755-8794-6-41 (PMC4015281; doi:10.1186/1755-8794-6-41)
Supplement: Additional file 1 — Contains supplementary materials and methods in addition to supporting data figures. [file 1755-8794-6-41-S1.pdf]

SUPPLEMENTARY MATERIALS AND METHODS.

Patient recruitment and sample collection

The study was approved by the human research ethics committee at University of Gothenburg, and oral and written informed consent was obtained from each volunteer before participation. Patients were selected from a consecutive cohort of patients undergoing gastroendoscopy at Sahlgrenska University Hospital, Gothenburg, during 2007 to 2009. These patients had been admitted to endoscopy due to dyspeptic symptoms, suspected occult bleeding or suspected malabsorption, and those giving written and oral consent to participate in the study were screened for *H. pylori* infection using a serology quick test (QuickVue *H. pylori* gII test, Quidel, San Diego, CA, USA) prior to endoscopy. Patients found positive in the test, and a small number of randomly selected patients found negative, were included in the study cohort. Patients had been fasted for a minimum of six hours before the examination and endoscopy was performed using a standard forward-viewing video gastroscope (Olympus GIF Q160; Olympus optical Co., Tokyo, Japan). From the included patients (n = 103), a serum sample and six biopsies from the antrum and corpus mucosa, respectively, were obtained during endoscopy. One antrum and one corpus biopsy was immediately placed in RNALater (Ambion-Invitrogen, Carlsbad, CA, USA) and stored at -70 °C for later mRNA analysis. Biopsies for protein extraction - one antrum and one corpus biopsy – were frozen and stored at -70 °C. Biopsies for *H. pylori* determination– one from antrum and one from corpus tissue – were placed in Portagerm transport medium (Biomérieux, Marcy l'Etoile, France) and later homogenized in 0.5 ml of culture media. They were thereafter cultured on Columbia ISO-A plates under microaerobic conditions (85% N_2, 10% CO_2, 5% O_2) at 37°C. *H.*

pylori colonies were identified according to colony morphology and biochemical analysis (urease test). Additional parallel biopsies, one antrum and one corpus biopsy, were fixed in formaldehyde for later histologic examination by the Pathology department at Sahlgrenska University Hospital. Serum samples were analyzed using in-house IgG and IgA ELISAs [1].

Patients who at subsequent examination were diagnosed with various bowel and other extragastric pathologies, including colitis or benign or malignant colorectal tumours, were excluded from the study (n = 21). The remaining patients were grouped into three groups; (1) patients without current *H. pylori* infection (Hp-; n = 37); (2) patients with current *H. pylori* infection but without corpus-predominant atrophic gastritis (Hp+; n = 34); (3) patients with current or past *H. pylori* infection and corpus-predominant atrophic gastritis (Atr; n = 7). For the purpose of this study, 6 and 8 patients were randomly selected from the Hp- and Hp+ groups, respectively. Patients were assigned to each group based on their histopathological scoring according to the Sydney system, in combination with their *H. pylori* culture results. Hp+ were culture positive in both corpus and antrum and Hp- negative in both locations. A patient was assigned to the corpus-predominant atrophy group if it had at least moderate atrophy in the corpus biopsy (score 2 or 3), concurrent with no or mild atrophy in the antrum biopsy (score 0 or 1), and positive *H. pylori* culture or serology.

RNA Extraction

The RNA from stomach samples was extracted from unfractionated biopsies of antrum and corpus tissue. The tissue was disrupted using a glass mortar and pestle

and added to a QIAshredder column (Qiagen, Hilden, Germany) before RNA extraction. Total RNA was extracted using the RNEasy Mini Kit (Qiagen), according to the instructions of the manufacturer. Thereafter, the extracted RNA was checked for integrity on a 1% agarose gel and the concentration was measured using a NanoDrop® (Thermo Scientific, Wilmington, DE). The extracted RNA was kept at -70˚C until further use.

Reference:

1. Mattsson A, Tinnert A, Hamlet A et al. Specific antibodies in sera and gastric aspirates of symptomatic and asymptomatic Helicobacter pylori-infected subjects. Clin Diagn Lab Immunol 1998;5:288-93.

Table S1. Forward and reverse primers used for qPCR analysis

Gene	Forward Sequence	Reverse Sequence
AMCase	AGGGCTACACTGGAGAGAACAG	GGTAGGGAATCCAACGATGAGC
ATP4b	ACAGACTCTCCACGCCTTCCTA	GCAGGAGAACTTGGTGTGGTTG
PGA5	TCTACTGCTCCAGTCTTGCCTG	TGGACAGTGTCGTATCCGAGGA
HPRT1	CATTATGCTGAGGATTTGGAAAGG	CTTGAGCACACAGAGGGCTACA

Supplementary Table S2. List of genes of subgroup g4 in Fig. 2.

			Hp-		Hp+		Atr	
ID	Symbol	Describtion	P.Value	Fold-diff[1]	P.Value	Fold-diff[1]	P.Value	Fold-diff[1]
ILMN_25328	AVPR1A	arginine vasopressin receptor 1A (AVPR1A)	0,054857	0,5530	0,242918	0,7045	0,581557	0,8494
ILMN_27662	DRD5	dopamine receptor D5 (DRD5)	1,45E-06	0,2920	0,001097	0,4944	0,268275	1,2366
ILMN_6484	AVPR1B	arginine vasopressin receptor 1B (AVPR1B)	0,468364	1,1256	0,628708	1,0818	0,461784	1,1276
ILMN_15738	ADRA1D	adrenergic, alpha-1D-, receptor (ADRA1D)	0,555523	0,9395	0,000303	0,6387	0,401537	1,0932
ILMN_11079	ENTPD2	ectonucleoside triphosphate diphosphohydrolase 2 (ENTPD2), transcript v	0,004957	1,8981	0,415351	1,1852	0,429204	1,1792
ILMN_11182	FGA	fibrinogen alpha chain (FGA), transcript variant alpha	6,17E-06	0,0502	7,08E-05	0,0846	0,416088	0,6588
ILMN_1134	P2RX1	purinergic receptor P2X, ligand-gated ion channel, 1 (P2RX1)	0,35449	0,8364	0,537124	0,8884	0,304966	0,8201
ILMN_138180	VWF	von Willebrand factor (VWF)	0,267451	0,6099	0,329466	0,6483	0,457317	0,7198
ILMN_13882	FGB	fibrinogen beta chain (FGB)	1,64E-08	0,0319	0,004679	0,2883	0,22246	0,6095
ILMN_139223	GP1BB	glycoprotein Ib (platelet), beta polypeptide (GP1BB)	0,867059	1,0930	0,816218	1,1314	0,427756	1,5284
ILMN_16715	PF4	platelet factor 4 (chemokine (C-X-C motif) ligand 4) (PF4)	0,138203	0,7467	0,942573	1,0139	0,728323	0,9354
ILMN_18231	F2	coagulation factor II (thrombin) (F2)	0,024547	0,6874	0,276823	0,8412	0,336034	0,8585
ILMN_21183	TREML1	triggering receptor expressed on myeloid cells-like 1 (TREML1)	0,14594	0,6907	0,084696	0,6418	0,959355	0,9874
ILMN_22394	PIK3CB	phosphoinositide-3-kinase, catalytic, beta polypeptide (PIK3CB)	0,329401	1,1863	0,06223	1,4004	0,454974	1,1391
ILMN_22820	CD40	CD40 antigen (TNF receptor superfamily member 5) (CD40), transcript va	0,270052	1,3505	0,987767	1,0041	0,558679	0,8541
ILMN_23651	IL11	interleukin 11 (IL11)	0,980529	1,0108	0,940765	0,9678	0,858889	1,0816
ILMN_25813	FGG	fibrinogen gamma chain (FGG), transcript variant gamma-A	9,22E-06	0,2759	0,00003	0,3087	0,750132	1,0746
ILMN_25961	F2R	coagulation factor II (thrombin) receptor (F2R)	0,671378	1,1778	0,93685	1,0310	0,440249	0,7414
ILMN_26128	COL3A1	collagen, type III, alpha 1 (Ehlers-Danlos syndrome type IV, autosomal do	0,73114	1,1473	0,596411	0,8088	0,20616	0,5977
ILMN_2685	CD40LG	CD40 ligand (CD40LG)	0,656798	0,9171	0,236691	1,2633	0,117141	0,7309
ILMN_27584	GP1BA	glycoprotein Ib (platelet), alpha polypeptide (GP1BA)	0,021047	0,7197	0,352433	0,8820	0,891233	1,0184
ILMN_28364	SAA1	serum amyloid A1 (SAA1), transcript variant 2	0,876349	1,1236	0,368132	0,5064	0,263166	0,4272
ILMN_3251	P2RY12	purinergic receptor P2Y, G-protein coupled, 12 (P2RY12), transcript variar	0,27246	0,8166	0,124491	1,3332	0,630984	0,9161
ILMN_3991	ADAMTS13	ADAM metallopeptidase with thrombospondin type 1 motif, 13 (ADAMTS	0,138632	0,8269	0,654588	1,0577	0,381997	1,1166
ILMN_4248	GP6	glycoprotein VI (platelet) (GP6)	0,105332	1,2401	0,093647	0,7999	0,078764	0,7906
ILMN_4337	BLOC1S3	biogenesis of lysosome-related organelles complex-1, subunit 3 (BLOC1S3	0,652836	0,9080	0,063396	1,5134	0,020624	0,5892
ILMN_4382	GNA13	guanine nucleotide binding protein (G protein), alpha 13 (GNA13)	0,00445	1,6581	0,036638	1,4257	0,657216	1,0742
ILMN_4441	PLSCR1	phospholipid scramblase 1 (PLSCR1)	0,052171	1,7148	0,617928	1,1420	0,938222	1,0208
ILMN_4953	CD9	CD9 molecule (CD9)	0,094629	1,8398	0,033341	2,2096	0,391226	1,3567

ILMN_6469	IL6	interleukin 6 (interferon, beta 2) (IL6)	0,88145	1,0785	0,406883	1,5273	0,968924	1,0199
ILMN_7767	ENTPD1	ectonucleoside triphosphate diphosphohydrolase 1 (ENTPD1)	0,461236	1,2858	0,484036	1,2695	0,250302	0,6731
ILMN_19861	FGL2	fibrinogen-like 2 (FGL2)	0,349577	1,4546	0,488205	1,3184	0,191604	0,5895
ILMN_2104	FGL1	fibrinogen-like 1 (FGL1), transcript variant 4	0,472727	0,7731	0,303602	0,6899	0,464682	0,7694
ILMN_4516	FN1	fibronectin 1 (FN1), transcript variant 7	0,733262	1,0624	0,796159	1,0469	0,823642	1,0404
ILMN_4882	THBS1	thrombospondin 1 (THBS1)	0,586926	1,3717	0,260838	1,9380	0,680814	1,2699
ILMN_1289	PLAT	plasminogen activator, tissue (PLAT), transcript variant 1	0,891756	0,9538	0,440778	0,7634	0,500744	0,7903
ILMN_22727	NR2F2	nuclear receptor subfamily 2, group F, member 2 (NR2F2)	2,76E-05	0,4202	0,000193	0,4800	0,906327	0,9807
ILMN_28694	CDH2	cadherin 2, type 1, N-cadherin (neuronal) (CDH2)	2,21E-06	0,0621	0,000276	0,1517	0,908044	0,9505
ILMN_28724	EDN1	endothelin 1 (EDN1)	0,001092	0,2748	0,002234	0,3046	0,48763	0,7851
ILMN_7827	SELP	selectin P (granule membrane protein 140kDa, antigen CD62) (SELP)	0,52352	1,2814	0,775165	1,1169	0,216732	0,6147

[1] Average fold-difference between antrum and corpus samples of the patients of each group. Fold-diff < 1 means a lower expression in corpus.

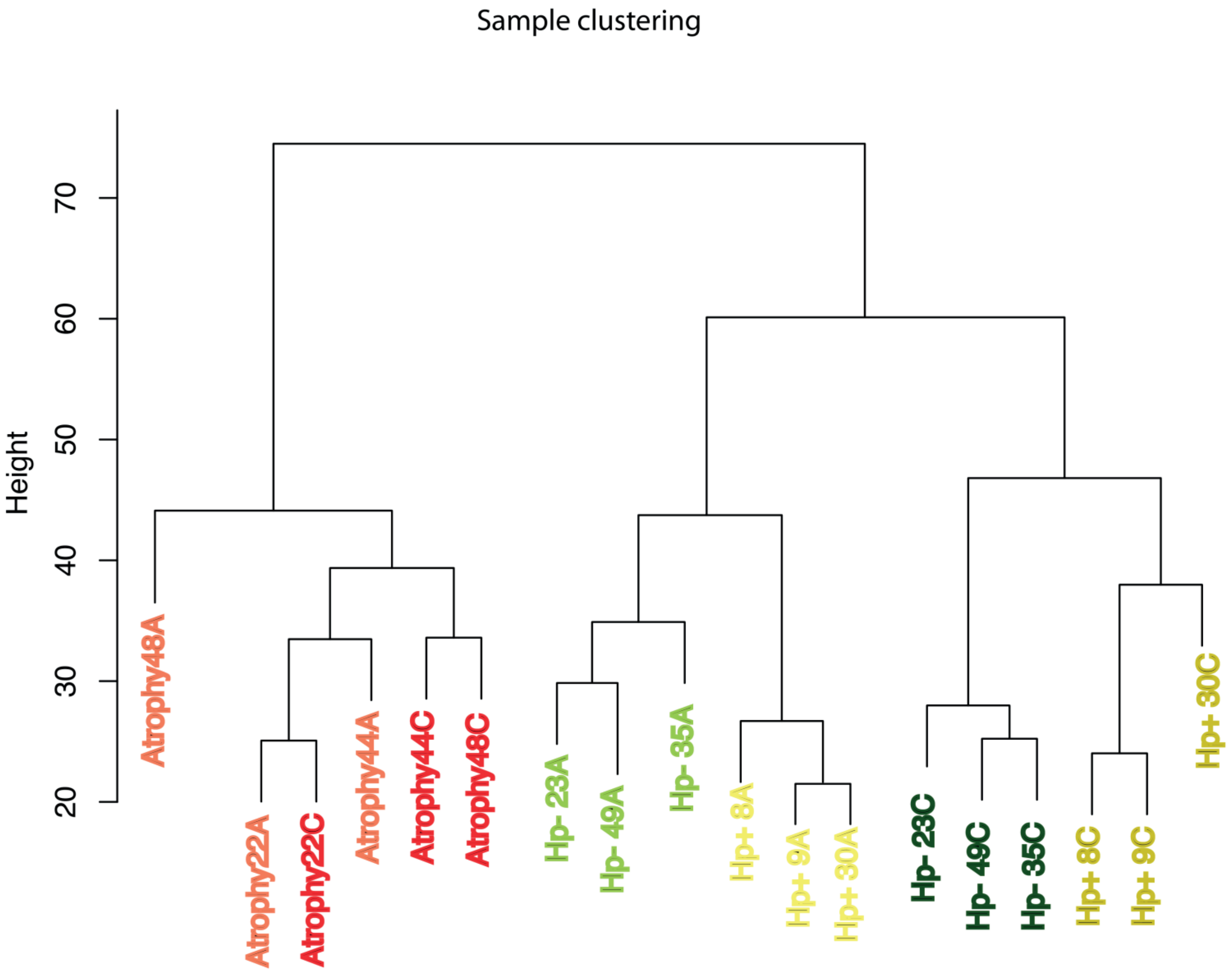

Supplementary Fig S1.
The microarray data from antrum and corpus samples of all the study subjects was subjected to unsupervised hierarchical clustering. Corpus biopsies are indicated by "C" , antrum biopsies by "A". The numbers indicate patient IDs. "Hp-" is *H. pylori*-uninfected subjects, "Hp+" is *H. pylori* infected subjects, and "Atrophy" is patients with corpus atrophy.

A. Corpus **B. Antrum**

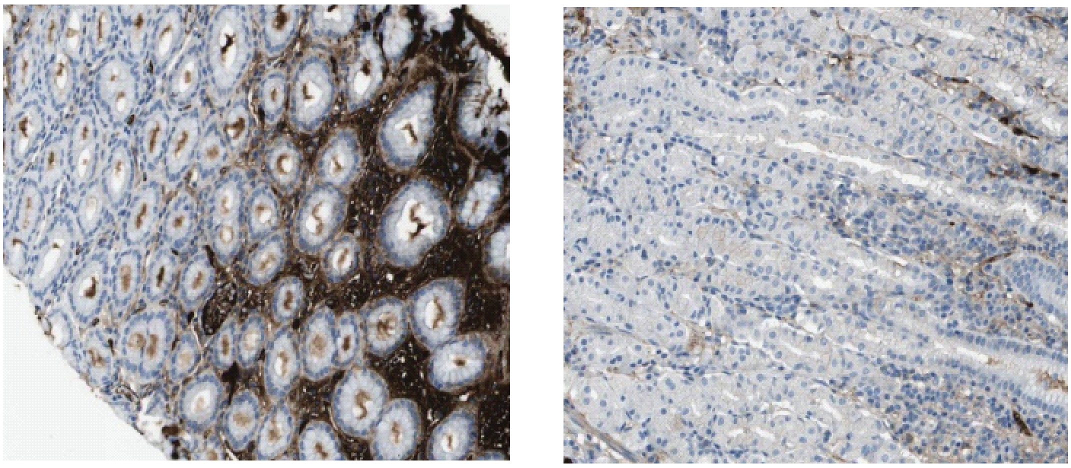

Supplementary Fig S2.
Images from Human Protein Atlas - www.proteinatlas.org. Corpus (A) and Antrum (B) mucosa from healthy individuals stained with antibodies to the alpha chain of fibrinogen (FGA), using the antibody CAB016776.

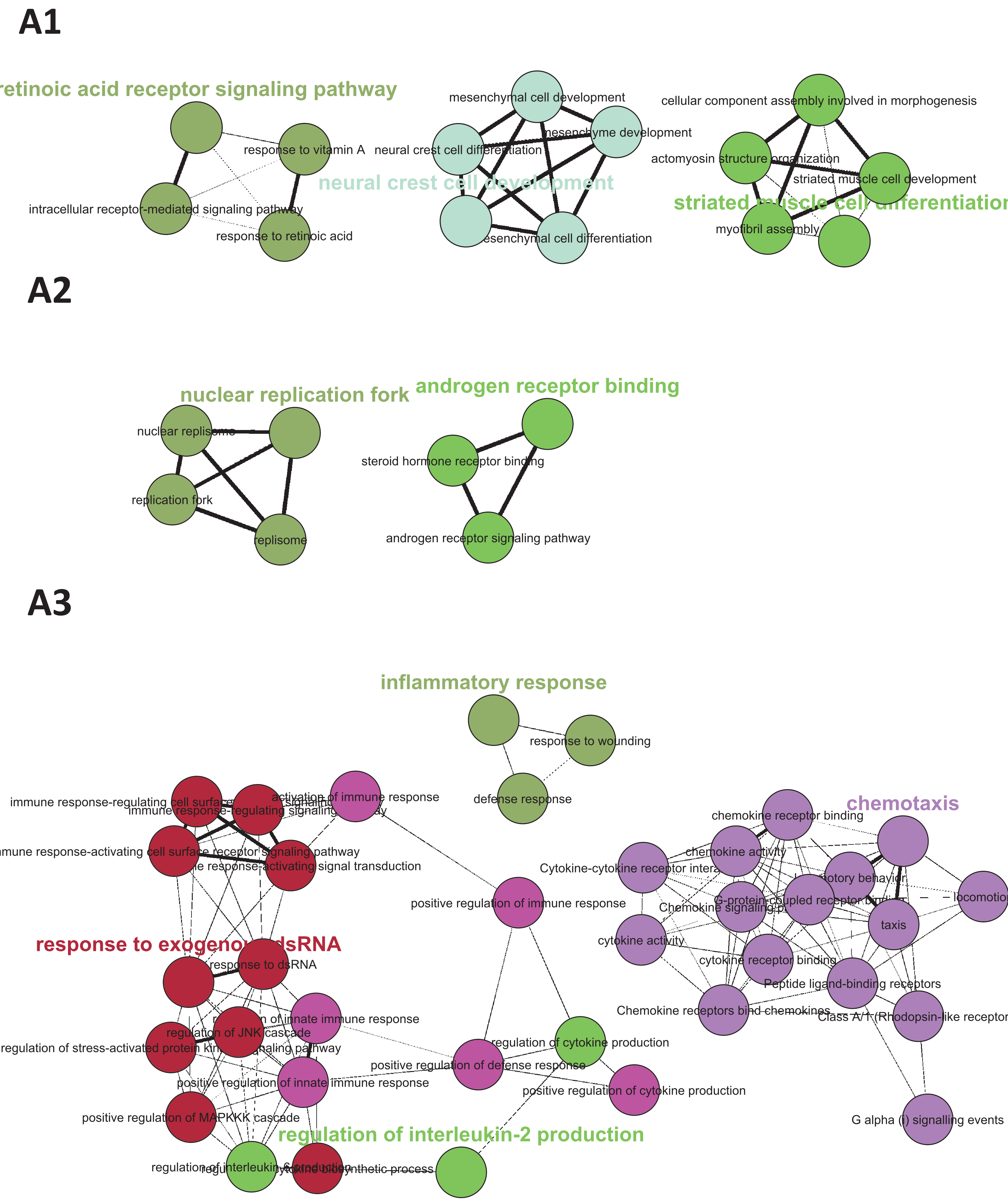

A1
retinoic acid receptor signaling pathway
response to vitamin A
intracellular receptor-mediated signaling pathway
response to retinoic acid
mesenchymal cell development
mesenchyme development
neural crest cell differentiation
neural crest cell development
mesenchymal cell differentiation
cellular component assembly involved in morphogenesis
actomyosin structure organization
striated muscle cell development
striated muscle cell differentiation
myofibril assembly
A2
nuclear replication fork
nuclear replisome
replication fork
replisome
androgen receptor binding
steroid hormone receptor binding
androgen receptor signaling pathway
A3
inflammatory response
response to wounding
defense response
activation of immune response
positive regulation of immune response
response to exogenous dsRNA
response to dsRNA
regulation of JNK cascade
positive regulation of innate immune response
positive regulation of MAPKKK cascade
regulation of cytokine production
positive regulation of defense response
positive regulation of cytokine production
regulation of interleukin-2 production
chemotaxis
chemokine receptor binding
chemokine activity
cytokine activity
cytokine receptor binding
taxis
locomotion
Peptide ligand-binding receptors
Chemokine receptors bind chemokines
G alpha (i) signalling events

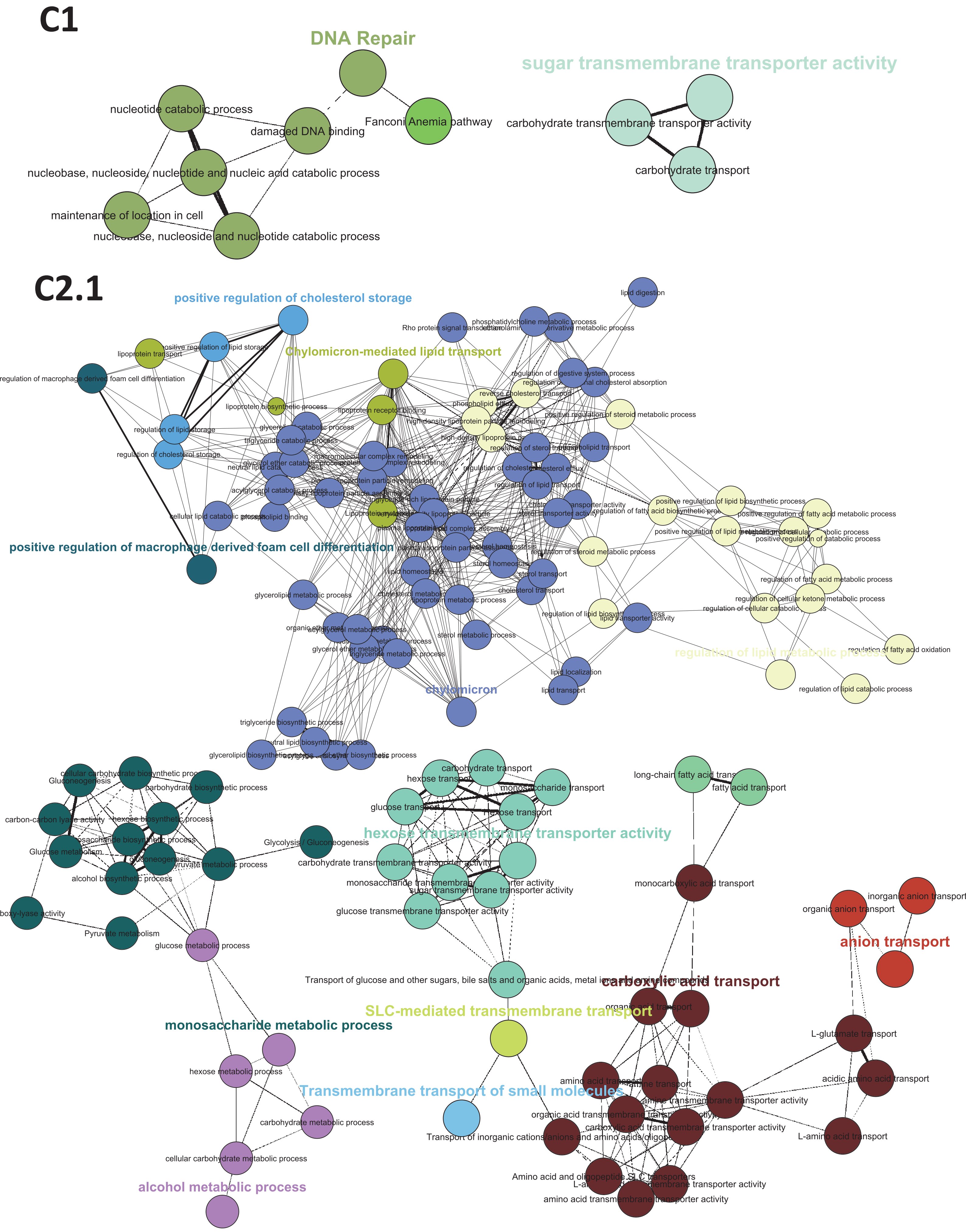
C1
DNA Repair
nucleotide catabolic process
damaged DNA binding
Fanconi Anemia pathway
nucleobase, nucleoside, nucleotide and nucleic acid catabolic process
maintenance of location in cell
nucleobase, nucleoside and nucleotide catabolic process
sugar transmembrane transporter activity
carbohydrate transmembrane transporter activity
carbohydrate transport
C2.1
positive regulation of cholesterol storage
lipid digestion
Rho protein signal transduction
phosphatidylcholine metabolic process
Chylomicron-mediated lipid transport
lipoprotein transport
regulation of macrophage derived foam cell differentiation
lipoprotein biosynthetic process
lipoprotein receptor binding
regulation of lipid storage
regulation of cholesterol storage
triglyceride catabolic process
macromolecular complex remodeling
protein-lipid complex remodeling
acylglycerol catabolic process
cellular lipid catabolic process
phospholipid binding
positive regulation of macrophage derived foam cell differentiation
regulation of digestive system process
reverse cholesterol transport
phospholipid efflux
high-density lipoprotein particle remodeling
positive regulation of steroid metabolic process
phospholipid transport
regulation of lipid transport
sterol transporter activity
positive regulation of lipid biosynthetic process
regulation of fatty acid biosynthetic process
positive regulation of fatty acid metabolic process
positive regulation of catabolic process
regulation of steroid metabolic process
sterol homeostasis
sterol transport
cholesterol transport
lipid homeostasis
glycerolipid metabolic process
lipoprotein metabolic process
regulation of fatty acid metabolic process
regulation of cellular ketone metabolic process
lipid transporter activity
acylglycerol metabolic process
sterol metabolic process
triglyceride metabolic process
regulation of lipid metabolic process
regulation of fatty acid oxidation
regulation of lipid catabolic process
lipid localization
lipid transport
chylomicron
triglyceride biosynthetic process
neutral lipid biosynthetic process
glycerolipid biosynthetic process
Gluconeogenesis
carbohydrate biosynthetic process
carbon-carbon lyase activity
hexose biosynthetic process
Glucose metabolism
pyruvate metabolic process
Glycolysis / Gluconeogenesis
alcohol biosynthetic process
Pyruvate metabolism
glucose metabolic process
carbohydrate transport
hexose transport
monosaccharide transport
glucose transport
hexose transmembrane transporter activity
carbohydrate transmembrane transporter activity
monosaccharide transmembrane transporter activity
sugar transmembrane transporter activity
glucose transmembrane transporter activity
long-chain fatty acid transport
fatty acid transport
monocarboxylic acid transport
inorganic anion transport
organic anion transport
anion transport
Transport of glucose and other sugars, bile salts and organic acids, metal ions and amine compounds
carboxylic acid transport
SLC-mediated transmembrane transport
organic acid transport
monosaccharide metabolic process
L-glutamate transport
hexose metabolic process
amino acid transport
acidic amino acid transport
amine transmembrane transporter activity
Transmembrane transport of small molecules
carbohydrate metabolic process
organic acid transmembrane transporter activity
carboxylic acid transmembrane transporter activity
L-amino acid transport
Transport of inorganic cations/anions and amino acids/oligopeptides
cellular carbohydrate metabolic process
Amino acid and oligopeptide SLC transporters
amino acid transmembrane transporter activity
alcohol metabolic process

C2.2

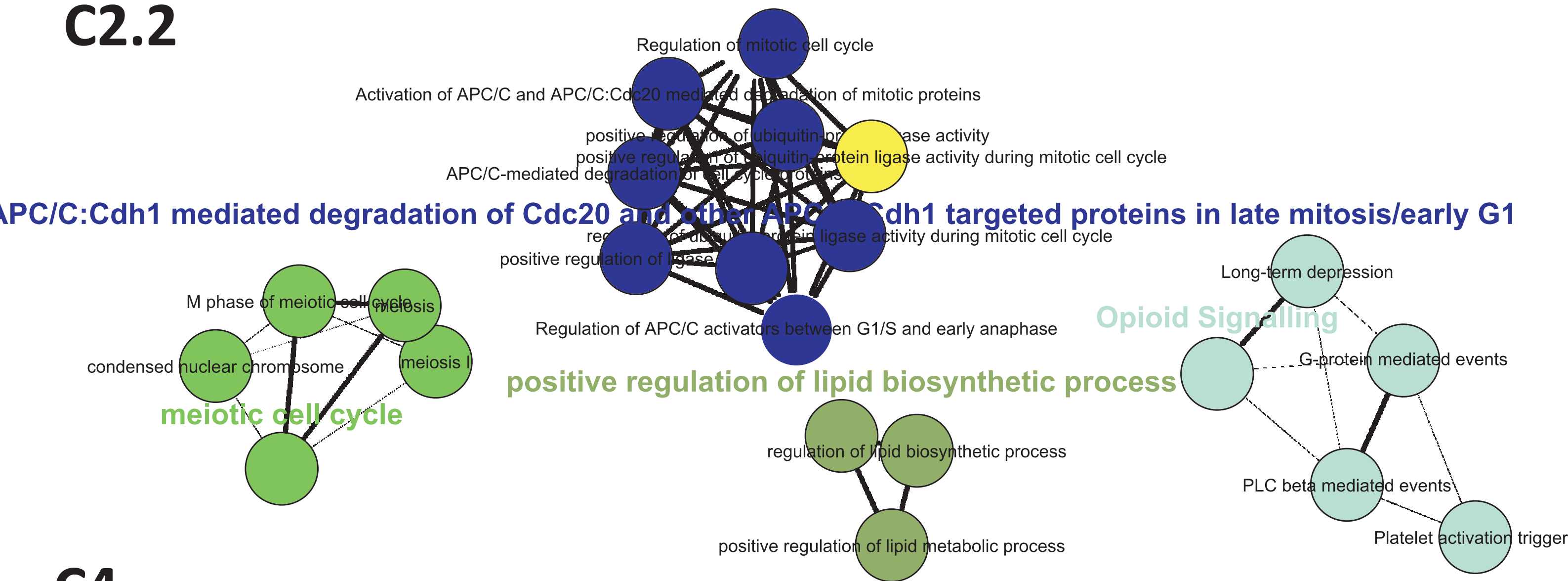
Regulation of mitotic cell cycle
Activation of APC/C and APC/C:Cdc20 mediated degradation of mitotic proteins
APC/C-mediated degradation of cell cycle proteins
APC/C:Cdh1 mediated degradation of Cdc20 and other APC/C:Cdh1 targeted proteins in late mitosis/early G1
positive regulation of ligase
Regulation of APC/C activators between G1/S and early anaphase
M phase of meiotic cell cycle
meiosis
condensed nuclear chromosome
meiosis I
meiotic cell cycle
positive regulation of lipid biosynthetic process
regulation of lipid biosynthetic process
positive regulation of lipid metabolic process
Long-term depression
Opioid Signalling
G-protein mediated events
PLC beta mediated events
Platelet activation trigger

C4

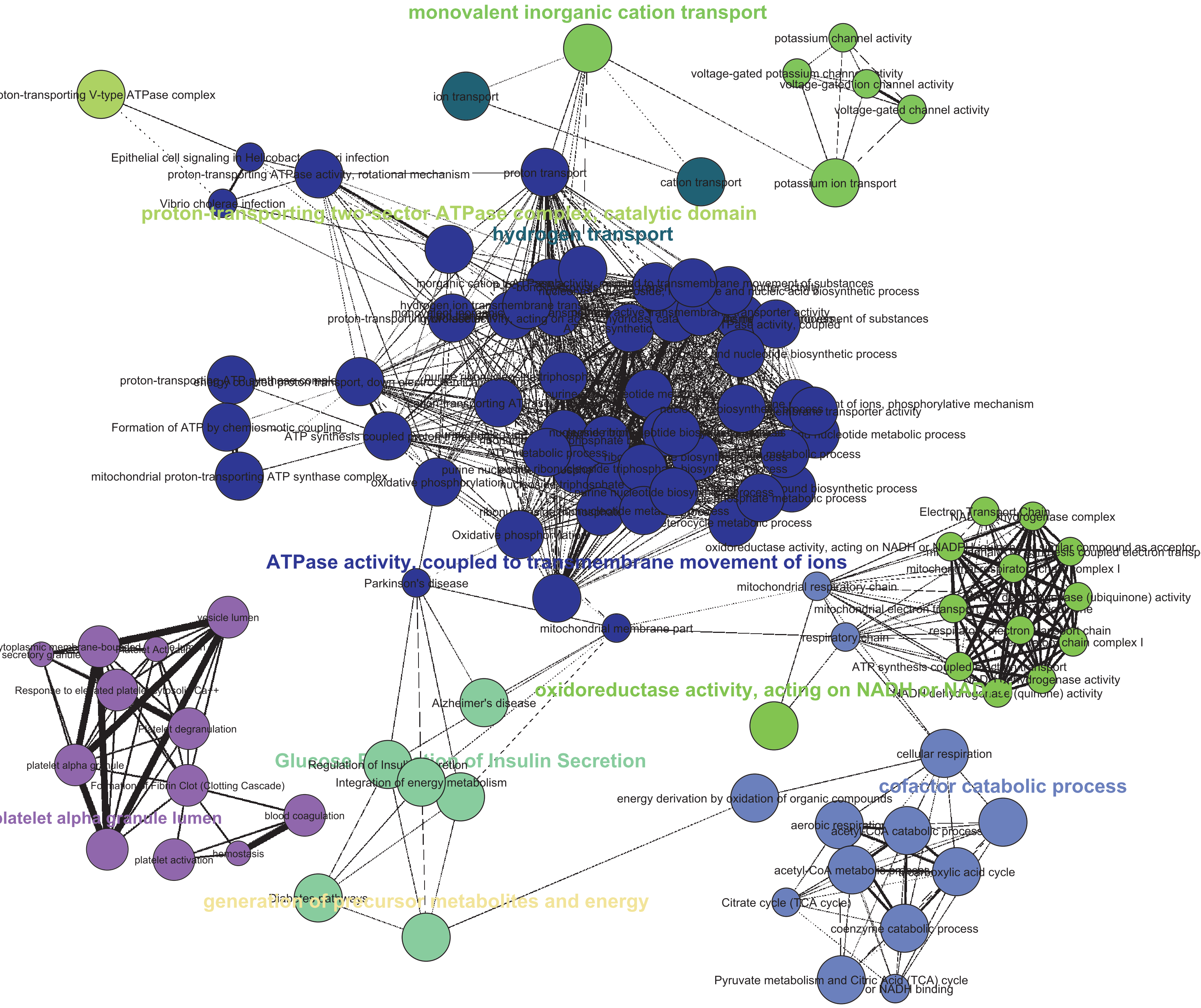
monovalent inorganic cation transport
potassium channel activity
voltage-gated potassium channel activity
voltage-gated ion channel activity
voltage-gated channel activity
potassium ion transport
ion transport
cation transport
proton-transporting V-type ATPase complex
Epithelial cell signaling in Helicobacter pylori infection
proton-transporting ATPase activity, rotational mechanism
proton transport
Vibrio cholerae infection
proton-transporting two-sector ATPase complex, catalytic domain
hydrogen transport
Formation of ATP by chemiosmotic coupling
ATP synthesis coupled proton transport
mitochondrial proton-transporting ATP synthase complex
oxidative phosphorylation
Oxidative phosphorylation
ATPase activity, coupled to transmembrane movement of ions
Parkinson's disease
mitochondrial membrane part
mitochondrial respiratory chain
mitochondrial electron transport
respiratory chain
Electron Transport Chain
NADH dehydrogenase complex
mitochondrial respiratory chain complex I
respiratory chain complex I
ATP synthesis coupled electron transport
oxidoreductase activity, acting on NADH or NADPH
NADH dehydrogenase (quinone) activity
Alzheimer's disease
Glucose Regulation of Insulin Secretion
Integration of energy metabolism
vesicle lumen
secretory granule
Response to elevated platelet cytosolic Ca++
Platelet degranulation
platelet alpha granule
Formation of Fibrin Clot (Clotting Cascade)
platelet alpha granule lumen
blood coagulation
platelet activation
hemostasis
generation of precursor metabolites and energy
cellular respiration
cofactor catabolic process
energy derivation by oxidation of organic compounds
aerobic respiration
acetyl-CoA catabolic process
acetyl-CoA metabolic process
tricarboxylic acid cycle
Citrate cycle (TCA cycle)
coenzyme catabolic process
Pyruvate metabolism and Citric Acid (TCA) cycle
NADH binding

Supplementary Figure S3 (above).

This is an enlargement of the ”bubbles”-part of Figure 3 in the main manuscript. The figure shows the significantly enriched ontologies associated to each cluster (C1-C4; A1-A3). Related ontologies are depicted by the same color, and are connected by lines. The names of the most highly significant ontologies are shown in larger font size.

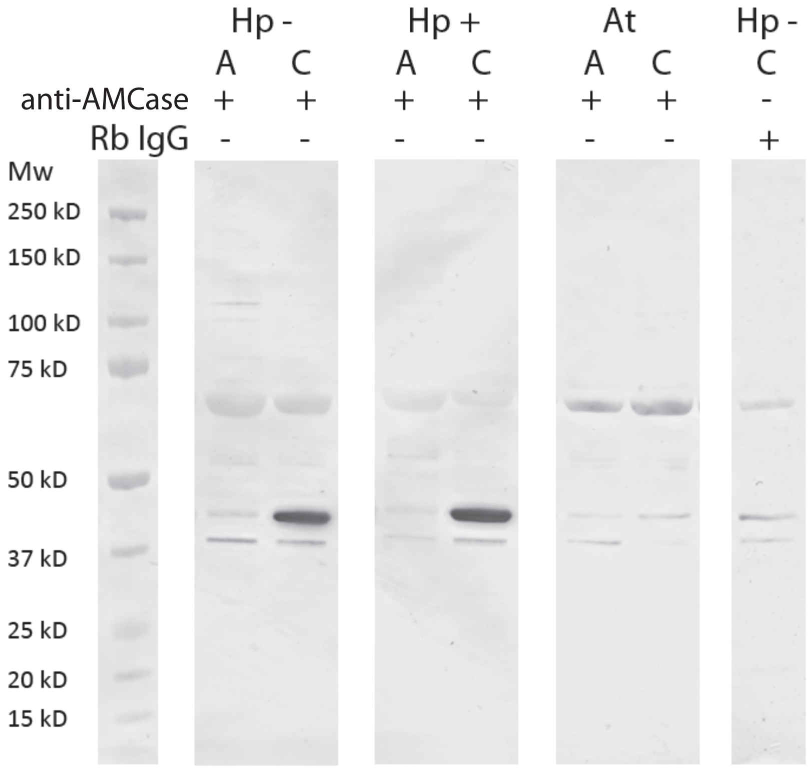

Supplementary Figure S4.
AMCase protein levels analysed with Western Blot in Antrum (A) and Corpus (C) samples of patients from Hp-, Hp+ and Atr groups. Blotting was performed using a polyclonal rabbit anti-AMCase antibody or rabbit IgG as negative control. A representative blotting image with one patient from each group is shown. The AMCase protein has a theoretical molecular weight of 49.9 kDa.

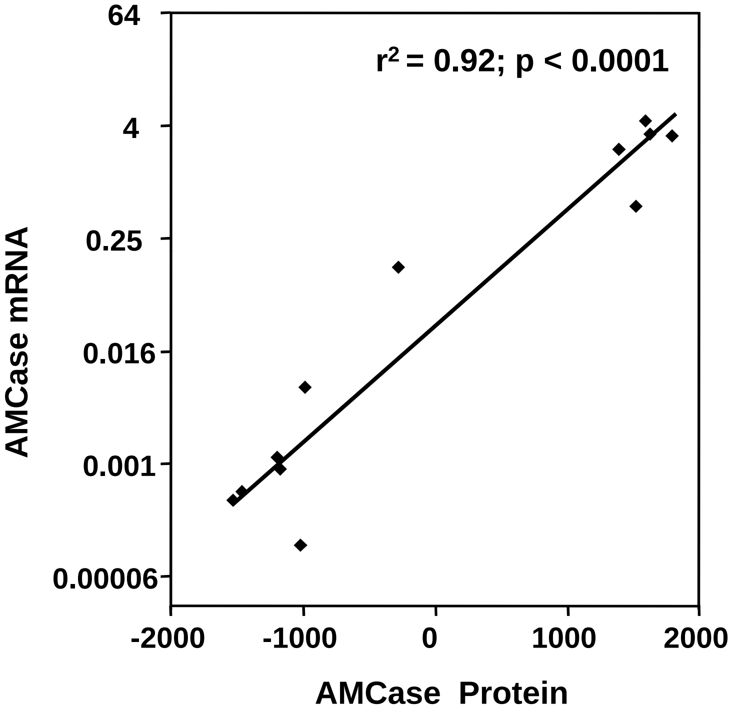

Supplementary Figure S5.
AMCase levels in antrum or corpus tissue of stomach samples, analyzed by RT-PCR (mRNA) and Western blot (protein). Biopsies analyzed for mRNA and protein content were collected at close distance from each other in the stomach mucosa. All samples in which both mRNA and protein were analysed in the same patient are included in the graph (n = 12 corpus/antrum samples from 6 patients, see Table S1 for details of patient analyses).
